# VEGF-Trap Modulates Retinal Inflammation in the Murine Oxygen-Induced Retinopathy (OIR) Model

**DOI:** 10.3390/biomedicines10020201

**Published:** 2022-01-18

**Authors:** Jesús Eduardo Rojo Arias, Vanessa Elisabeth Englmaier, József Jászai

**Affiliations:** 1 Department of Anatomy, Medical Faculty Carl Gustav Carus, Technische Universität Dresden, 01307 Dresden, Germany; vanessa_elisabeth.englmaier@tu-dresden.de; 2 Wellcome-MRC Cambridge Stem Cell Institute, Jeffrey Cheah Biomedical Centre, Cambridge Biomedical Campus, University of Cambridge, Cambridge CB2 0AW, UK

**Keywords:** Aflibercept, VEGF-Trap, microglia, retina, inflammation, oxygen-induced retinopathy, OIR

## Abstract

Anti-Vascular Endothelial Growth Factor (VEGF) agents are the first-line treatment for retinal neovascular diseases, which represent the most prevalent causes of acquired vision loss world-wide. VEGF-Trap (Aflibercept, AFL), a recombinant decoy receptor recognizing ligands of both VEGFR-1 and -2, was recently reported to be highly efficient in improving visual acuity and preserving retinal anatomy in individuals affected by diabetic macular edema. However, the precise molecular and cell biological mechanisms underlying the beneficial effects of this novel tool have yet to be elucidated. Using the mouse oxygen-induced retinopathy (OIR) model as a surrogate of retinopathies with sterile post-ischemic inflammation, such as late proliferative diabetic retinopathy (PDR), retinopathy of prematurity (ROP), and diabetic macular edema (DME), we provide evidence that AFL modulates inflammation in response to hypoxia by regulating the morphology of microglial cells, a parameter commonly used as a proxy for changes in their activation state. We show that AFL administration during the hypoxic period of OIR leads to an increased number of ramified Iba1^+^ microglial cells/macrophages while subsequently limiting the accumulation of these cells in particular retinal layers. Our results suggest that, beyond its well-documented beneficial effects on microvascular regeneration, AFL might exert important modulatory effects on post-ischemic retinal inflammation.

## 1. Introduction

Retinopathy of prematurity (ROP), diabetic retinopathy (DR), and neovascular age-related macular degeneration (AMD), represent the most prevalent causes of acquired vision loss in industrialized countries. Jointly known as ischemic retinopathies, these pathologies are characterized by over-activation of the Vascular Endothelial Growth Factor (VEGF) pathway due to an imbalance between metabolic demand and oxygen supply in the retina and affect individuals within all age cohorts, from pre-term newborns to the elderly population [1,2,3,4,5,6]. Typical symptoms resulting from these conditions include vascular leakiness, compensatory uncontrolled angiogenesis, neuronal dysfunction, and microglial cell activation [7,8,9,10]. Indeed, antigen-presenting cells are recruited to the retina in response to blood–retinal barrier disruptions [11], with inflammatory cells further promoting vascular hyper-permeability [12]. In this context, diabetic retinopathy (DR) was originally referred to as “diabetic retinitis” [13] in allusion to its characteristic inflammatory nature [14,15].

Microglial cells are sentinels of the immune system, which, in the retina, reside primarily in the plexiform layers flanking the inner nuclear layer, with a few others found between nerve fibers [16,17]. While microglial cells exhibit a ramified morphology in the murine retina under healthy physiological conditions [9,17], they migrate within the tissue during the phase of sterile post-ischemic inflammation of the oxygen-induced retinopathy protocol (OIR) [18]. Notably, previous studies indicate that this process might be of central relevance to retinal neovascularization [19]. Additionally, the dramatic morphological changes undergone by microglial cells and the massive increase in their numbers as a consequence of hypoxic stress and post-ischemic inflammation in the OIR retina might also play an important role in the elimination of pathological neovascular tufts and in the promotion of ordered revascularization (vascular regeneration) [20,21].

Overall, however, the origin and function of microglial cells in the central nervous system (CNS) in different contexts remains only partially understood. Pro-inflammatory macrophages in the circulation, for instance, are known to be attracted to CNS lesion sites under pathological conditions [22,23]. Nevertheless, recent studies using genetically labeled cells suggest that reactive microglial cells have their origin within the retina in the OIR model [24]. However, while changes in the density of microglial cells in response to ischemia have been described in various studies [9,19,25], reports of changes in the localization of reactive microglial cells within the OIR retina are inconsistent, with outcomes being apparently dependent on the particular strain of the examined mice [19,21,26].

The importance of myeloid cells for the retinal vasculature lies in their capacity to secrete pro-angiogenic factors, which, in excess, might drive diverse ocular pathologies, including edema and neovascularization in ischemic retinopathies [27]. Thus, the use of therapeutic agents with the capacity to regulate the activation status of microglia/macrophages might be beneficial in the clinical setting to hinder the emergence and progression of these pathologies and to normalize the retinal microvascular architecture. Notwithstanding, most of the anti-angiogenic tools currently in use are incapable of repairing or preventing these disturbances [10,28,29]. In this sense, anti-VEGF-A monotherapies constitute the first-line treatment for diabetic retinal complications. However, pharmacological down-regulation of VEGF-A is known to result in the compensatory up-regulation of alternative factors, including the placental growth factor (PlGF). Notably, concomitant inhibition of PlGF and VEGF-A, either systemically or locally, was recently shown to not only reduce neovascularization and vascular leakage but also to modulate accompanying inflammatory responses in murine models of ocular neovascularization [30,31,32,33]. In agreement, the blockade of VEGFR1, the cognate receptor for PlGF, was shown to have similar effects in the OIR retina, while the disruption of either VEGF-A or VEGFR-2 had a modulatory effect on inflammation in these models [30,31,33,34]. In agreement with this notion, we have previously shown that systemic administration of VEGF-Trap (Aflibercept, henceforth AFL) efficiently counteracts neovascularization, promotes vascular regeneration, and reduces vascular leakiness while improving visual circuit functionality [8]. Here, we report that AFL additionally affects the morphology, density, and distribution of microglial cells in the OIR retina.

## 2. Materials and Methods

### 2.1. Ethics Statement and Animal Care

Animal experiments were performed in strict accordance with German Animal Welfare legislation. Mouse experiments were approved by animal welfare authorities of the Saxonian Government (Landesdirektion Sachsen). Animals were handled and housed in a 12-h light/dark cycle with water and food ad libitum according to the German Federal guidelines for the use and care of laboratory animals. All efforts were made to minimize animal suffering. Prior to eye collection, mice were sacrificed by anesthesia overdosing, followed by cervical dislocation. All mice were acquired from Charles River Laboratories (Sulzfeld, Germany), either to breed in-house or as pregnant females. Newborns were allocated to experimental groups irrespective of their sex. All experimental protocols (TVV26/2017) were checked by the Institutional Animal Welfare Officer (Tierschutzbeauftragter) of the Dresden University of Technology and were subsequently approved by the Landesdirektion Dresden with the approval number DD24.1-5131/394/ issued on 29 June 2017. The study was not pre-registered.

### 2.2. Oxygen-Induced Retinopathy and Aflibercept Treatment

To induce pathologic neovascularization in the retina, we used a modified version of the oxygen-induced retinopathy protocol [18]. Briefly, C57BL/6J mouse pups and their nursing mothers were exposed to 75% oxygen from P6 to P11 (P0 = birthdate); before and afterwards, all mice stayed in ambient air and were kept under normal husbandry conditions (as described above). AFL (kindly provided by Bayer AG, Leverkusen, Germany) was diluted in sterile pyrogen-free Dulbecco’s PBS (Sigma–Aldrich, Munich, Germany) and administered i.p. to half the pups in each litter at a dose of 25 mg/kg body weight on P13 and P15 for animals sacrificed at P17. Experimental mice subjected to the OIR protocol belonging to the AFL-treated cohort and sacrificed at P19 received AFL either on days P13 and P15 (OIR+AFL×2) or on days P13, P15, and P17 (OIR+AFL×3). Non-injected OIR littermates and age-matched mice kept under normoxic conditions were used as controls.

### 2.3. Tissue Collection and Fixation

Prior to tissue collection, mice were deeply anesthetized by an intra-peritoneal injection of ketamine (300 mg/kg BW; Ratiopharm, Ulm, Germany) and xylazine (30 mg/kg BW; Ratiopharm, Ulm, Germany) and sacrificed by cervical dislocation. Thereafter, eyes were promptly removed from their orbits and fixed by immersion in 4% paraformaldehyde (PFA; Sigma-Aldrich, Munich, Germany) in 0.1M PBS buffer pH 7.4 (Merck Millipore, Darmstadt, Germany) at 4 °C for 4 h. Subsequently, samples were further processed for analysis.

### 2.4. Paraffin Embedding

Fixed eyes were incubated overnight in 70% ethanol at 4 °C prior to dehydration in a graded ethanol series. Samples were then cleared with xylene (RotiClear; CarlRoth, Karlsruhe, Germany), followed by incubation in a paraffin bath for 90 min at 64 °C. Molds were used to cast paraffin blocks containing eye samples oriented with the optic nerve-cornea axis parallel to the surface. Sections of 4 µm thickness were longitudinally sectioned on a rotating microtome (Leica Jung RM 2065; Leica Microsystems, Wetzlar, Germany) and mounted on microscopic slides (Thermo Fisher Scientific, Waltham, MA, USA).

### 2.5. Immunofluorescent Staining of Paraffin Sections

Paraffin sections were deparaffinized by three 5-min rounds of immersion in xylene and subsequently rehydrated through sequential incubations in a series of graded ethanol solutions followed by distilled water. Antigen unmasking prior to immunohistochemical detection was performed by boiling slides in citrate buffer pH 6 (citric acid, monohydrate/sodium citric acid, dihydrate; Merck Millipore, Darmstadt, Germany) twice for 5 min. Slides were then washed 3 × 10 min in PBS and blocked for 1 h in 10% Normal Goat Serum in PBS (NGS 10%; Vector Laboratories, Burlingame, CA, USA). Immunolabeling was performed by incubating the slides overnight at 4 °C with an antibody against Iba-1 (Cat. Nr. 019-19741, Wako Chemicals, Neuss, Germany) diluted 1:250 in NGS 10%. Unbound primary antibodies were removed by washing the slides 3 × 10 min in PBS at room temperature (RT). Antigen–antibody complexes were detected by incubating slides for 1 h at RT with an Alexa Fluor 546-conjugated goat anti-rabbit secondary antibody (Cat. Nr. A-11035, ThermoFisher Scientific, Eugene, OR, USA) diluted 1:1000 in NGS 10%. Cell nuclei were labeled with DAPI (1 µg/mL; Molecular Probes, Eugene, OR, USA). After 3 × 10 min of washing with PBS, stained sections were mounted with Mowiol (Sigma–Aldrich, Munich, Germany) and coverslipped.

### 2.6. Retinal Cup Immunofluorescent Staining and Flat-Mounting

After eyes were fixed in 4% PFA (as described above), retinal cups were isolated, washed with ice-cold PBS, and washed twice with PBS 0.1% Triton X-100 (PBSTX-0.1%). After two 15-min permeabilization steps with PBSTX-1%, retinas were washed 3 × 10 min with PBSTX-0.1% and blocked for 1 h with PBSTX-0.1% NGS 10%. Subsequently, retinas were incubated overnight at 4 °C with primary antibodies diluted in PBSTX-0.1% NGS 10%. The following morning, retinas were washed 3 × 10 min in PBSTX-0.1% and incubated for 1 h at room temperature with fluorochrome-conjugated antibodies in PBSTX-0.1% NGS 10%. After three 10-min washes with PBSTX-0.1%, retinas were flattened by four radial incisions, transferred onto microscopic slides, flat-mounted in 30% glycerol in PBS with the ganglion cell layer upwards, and coverslipped. The primary antibodies were rabbit FITC-conjugated anti-CD146 (clone ME-9F1, dilution 1:200, Miltenyi Biotech, Cat. Nr. 103-102) and anti-Iba1 (as before). The secondary antibodies (all 1:1000) were Alexa Fluor 488-conjugated goat anti-rat (Cat. Nr. A-11006, ThermoFisher Scientific, Eugene, OR, USA) and Alexa Fluor 546-conjugated goat anti-rabbit secondary (as before).

### 2.7. Microscopy and Morphometric Analyses

Images of immuno-fluorescently stained whole-mounts and cross-sections were acquired in an Axio Observer.Z1 microscope (Carl Zeiss Microscopy, Jena, Germany). Overviews of complete retinal whole-mounts and cross-sections were generated by acquiring adjacent images with the MosaiX function and merging them with the Stitching tool of AxioVision Rel. 4.9.1 (Carl Zeiss Microscopy, Jena, Germany). Subsequently, the numbers of round, amoeboid, and ramified Iba1^+^ cells were determined using the multi-point tool of ImageJ [35] in overview images of entire immunolabeled retinal flat mounts. Similarly, Iba1^+^ cells were quantified using the multi-point tool of ImageJ in entire retinal cross sections within the following layers: ganglion cell layer (GCL), inner plexiform layer (IPL), inner nuclear layer (INL), outer plexiform layer (OPL), and outer nuclear layer (ONL). To determine the density of Iba1^+^ cells per retina, the total number of cells and the number of cells per layer were divided by the area of the retinal cup, as measured in ImageJ.

### 2.8. Statistical Analysis

Statistical analyses were performed in GraphPad Prism (Version 9.1.1, GraphPad Software LLC, San Diego, CA, USA) using one-way ANOVA with Tukey’s Multiple Comparison test. *p*-values < 0.05 were considered statistically significant.

## 3. Results

### 3.1. AFL Administration Increases the Number of Ramified Microglial Cells in the OIR Retina

**Figure 1:**
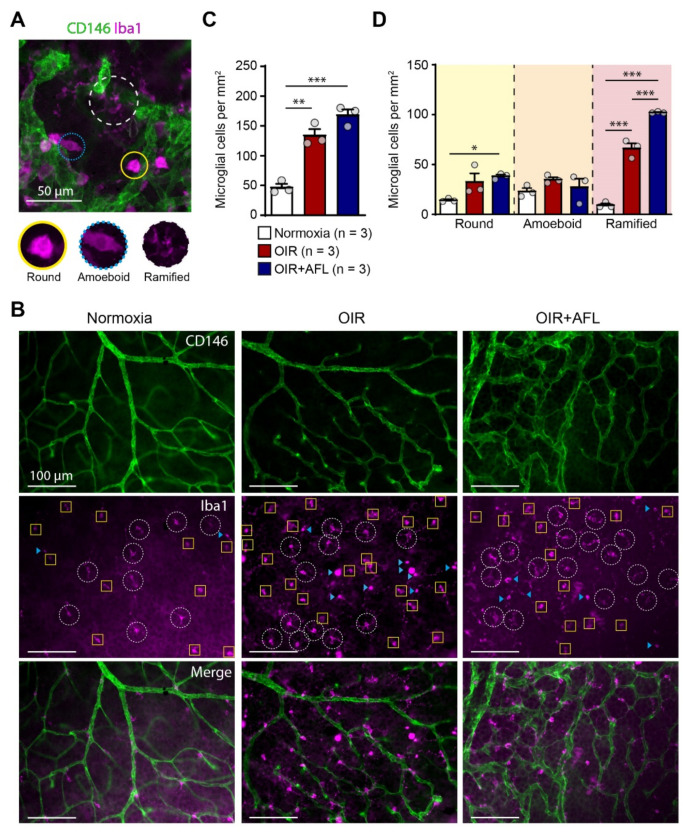
The proportion of ramified macrophage/microglial cells increases in retinas from OIR mice upon AFL treatment. (**A**) Representative micrograph of a CD146 (green) and Iba1 (magenta) immuno-fluorescently labeled retina from a P17 OIR+AFL mouse. CD146-positive structures in the image are neovascular tufts. Iba1^+^ cells were morphologically discriminated into round (solid line, yellow), amoeboid (dotted line, blue), and ramified (dashed line, white). Scale bar: 50 μm. (**B**–**D**) Iba1^+^ cells were quantified according to their morphology in immuno-fluorescently labeled flat-mounted retinas from P17 normoxic (white), OIR (red) and OIR+AFL (blue) mice (n = 3 per treatment). Scale bar: 100 μm. In (**B**), ramified cells are encircled by a white dashed line, round cells are labelled with a blue arrowhead, and amoeboid cells are enclosed in yellow squares. The images are representative of vascularized regions free of neovascular tufts. In graphs, the total number of Iba1^+^ cells (**C**), irrespective of their morphology, as well as the number of cells exhibiting each morphology (**D**), are shown as quantified in entire retinal flat-mounts. Error bars display +1 S.E.M. n.s., non-significant; * *p* < 0.05; ** *p* < 0.01, *** *p* < 0.001.

Microglial cells have been linked to pathological retinal angiogenesis [19,25,36,37]. Thus, we decided to explore the response of this cell population to the OIR protocol and to AFL administration. For this purpose, flat-mounted P17 retinas were immuno-labeled with an antibody recognizing Iba1, i.e., the ionized calcium binding adaptor molecule 1. Iba1 is a marker of resident microglial cells and of cells of monocytic origin entering tissues during inflammatory reactions [38]. Retinal Iba1-positive cells were categorized according to their morphology into round, amoeboid, and ramified cells (Figure 1A) and quantified in the entire retinal tissue (Figure 1B–D). AFL-treated retinas displayed an increased number of ramified microglia (classically defined as “resting,” possibly pro-regenerative) at P17 relative to OIR non-injected littermates (Figure 1D). However, compared to non-injected OIR littermates, AFL administration had no effect on the number of (activated) round or amoeboid cells, and the number of round Iba1^+^ cells relative to normoxic controls remained significantly higher in retinas from AFL-treated mice (Figure 1D). Similarly, the total number of Iba1^+^ cells was significantly higher in both OIR and OIR+AFL retinas than in normoxic age-matched controls (Figure 1C).

### 3.2. Stratification and Density of Iba1^+^ Cells in Retinal Cryosections of P19 Mice

**Figure 2:**
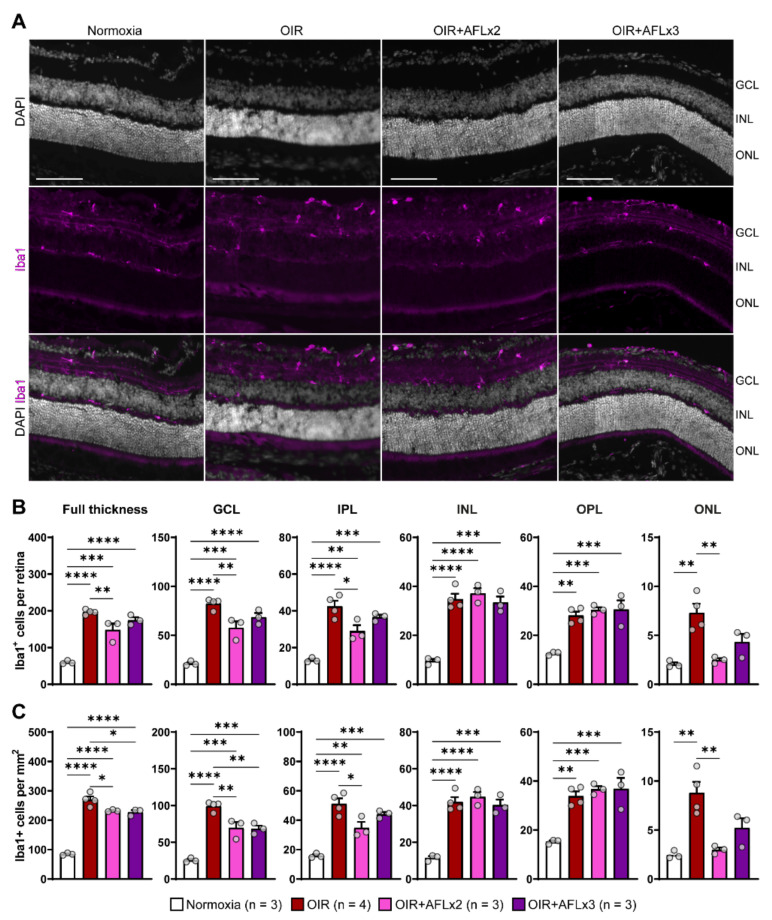
Immunofluorescent labeling and quantification of Iba1^+^ cells in retinal cross sections. (**A**) Representative micrographs of Iba1 (magenta) immuno-fluorescently labeled retinal cross-sections from P19 Normoxic, OIR, OIR+AFLx2, and OIR+AFLx3 mice. DAPI counterstain is shown in greyscale. GCL, ganglion cell layer; INL, inner nuclear layer; ONL, outer nuclear layer. Scale bar: 100 µm. (**B**,**C**) Total numbers (**B**) and density (**C**) of Iba1^+^ cells in the full retina and in distinct retinal layers. IPL, inner plexiform layer; OPL, outer plexiform layer. Error bars display +1 S.E.M. * *p* < 0.05; ** *p* < 0.01, *** *p* < 0.001, **** *p* < 0.0001.

Iba1^+^ microglial cells residing in the retina provide complete surveillance of the tissue and, during inflammation, can be rapidly activated and migrate between layers to support the elimination of large amounts of cellular debris. Immunofluorescent labeling of retinal cross sections from P19 normoxic, OIR, OIR+AFLx2, and OIR+AFLx3 mice revealed high numbers of Iba1^+^ cells in distinct retinal layers, including the ganglion cell layer (GCL), the inner and outer plexiform layers (INL and OPL), and the inner nuclear layer (INL) (Figure 2A). The number and density of these cells, however, was significantly higher in retinas of animals subjected to the OIR protocol, irrespective of AFL treatment, compared to those of normoxic controls (Figure 2B,C). A more detailed analysis taking into account the location of Iba1^+^ cells showed a significant reduction in their number within the GCL of OIR animals that received either two or three doses of AFL and a reduction in their density within this layer in OIR+AFLx2 retinas (Figure 2B,C). Two doses of AFL also led to a significant reduction in the number and density of Iba1^+^ cells within the IPL and outer nuclear layer (ONL) compared to retinas of OIR animals that were not treated with AFL (Figure 2B,C). Notably, a trend towards a similar pattern was observed in the ONL of OIR+AFLx3 retinas, although this difference was non-significant (Figure 2B,C). Moreover, the number and density of Iba1^+^ immunoreactive cells within the ONL of OIR+AFLx2 and OIR+AFLx3 retinas were not significantly different from those detected in normoxic controls. Similarly, no significant differences in the number or density of Iba1^+^ cells were detected within the OPL of OIR+AFLx2 and OIR+AFLx3 retinas compared to those of OIR mice, although both of them were higher in all of these conditions compared to normoxic controls.

## 4. Discussion

The types and roles of microglial cells remain both a topic of active debate among the scientific community. In health, the processes of ramified microglial cells are in constant contact not only with neurons and astrocytes but also with blood vessels so as to monitor their functional state. However, microglial functions are known to be highly context-dependent [22,39,40]. In response to laser-induced microlesions, for instance, the processes of ramified microglial cells are directed towards injury sites, where they phagocytose damaged tissue [39]. Furthermore, microglia are also known to limit inflammation in certain scenarios [41]. In this context, retinal damage associated to diabetic retinopathy has been linked to low-level chronic inflammation and tightly correlates with the appearance of microvascular abnormalities [42]. In this sense, it is possible that the increased number of ramified microglial cells we observed in AFL-treated OIR retinas at P17 may either regulate inflammation or support the clearance of increased levels of cellular debris as a consequence of hypoxic damage to the retinal tissue. Alternatively, Iba1^+^ cells might be involved in modulating the permeability of the blood-retinal-barrier by phagocytosing astrocytic end-feet, in sealing and repairing it when damaged, and/or in regulating vascular tone and blood flow, as recently shown in diverse studies in the brain [43,44,45]. Given that our previous work revealed a significant mopho-functional recovery of the retina [8], we hypothesize that the increased number of ramified cells might support the elimination of pathological tufts. Overall, however, additional studies aimed at shedding light on the function of ramified microglial cells in the retinal tissue of OIR mice are required.

In hyperglycemic conditions, retinal microglial cells have been reported to secrete molecules that cause highly localized low-level inflammation [46,47]. Similarly, in ischemic and hypoxic conditions, inflammatory cells contribute significantly to the “angiogenic switch,” as observed in tumor angiogenesis and neovascular eye diseases [20,48]. In the eye, inflammatory cells secrete a variety of cytokines (e.g., Tumor Necrosis Factor α [TNF-α]), and their accumulation is a distinguishing feature of pathological angiogenesis [21]. While measuring cytokine levels during the different stages of the OIR protocol and in response to AFL could be of relevance, potential changes in the expression kinetics of these molecules have yet to be further investigated.

In the OIR model, the density of microglial cells changes dramatically during both the period of hyperoxia and, in agreement with our observations, the phase of relative hypoxia [9,19,25,46,49,50], with the predominant myeloid cell population in areas of retinal neovascularization having been previously reported to originate from proliferating retinal microglia [24]. These findings stand in sharp contrast with earlier reports describing minimal proliferation of microglial cells within this tissue [9,51]. Whether the Iba1^+^ cells we detected in the murine retina originate from resident microglial cell proliferation or were recruited to the tissue from extraretinal sources remains an open question.

**Figure 3:**
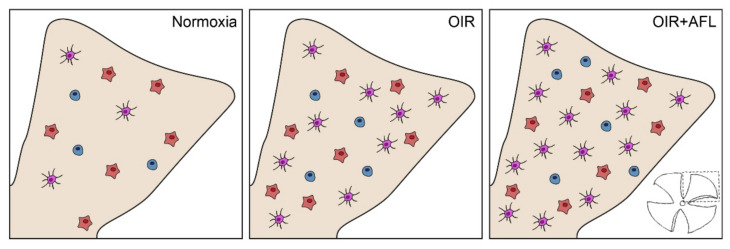
Schematic representation of Iba1+ cells in P17 flat-mounted normoxic, OIR and OIR+AFL retinas. Round cells are shown in blue, amoeboid in orange, and ramified in purple. One of the four quadrants in which retinal tissues were cut for flat-mounting (bottom right) is schematically shown in beige for each condition (Normoxia, OIR and OIR+AFL).

In the OIR model, the increased counts of monocytic and T cells in neovascularization fronds (Figure 3) are clear evidence of hypoxia/ischemia-induced inflammation [24,51,52,53]. However, previous reports suggest that the activation status of microglial cells might have a more profound effect than their numbers on vascular regeneration [21]. Indeed, the modulation of macrophage polarization has been shown to be sufficient for inducing vessel normalization in tumors [54], with M2-polarized cells linked to the promotion of retinal neovascularization [55]. Thus, although these cells are necessary to fulfill certain physiological functions, such as debris clearance, when present in elevated numbers they can drive the excessive release of cytokines. Hence, a tight control of their numbers and activation status might both represent potential targets for mitigating pro-inflammatory responses while promoting blood vessel normalization [56,57]. Given their heterogeneity [58], therapeutic agents could be targeted solely to reactive retinal microglial cells [59], such as IL-34-dependent IPL-resident ones [60] or FGF-2-releasing retinopathy-promoting ones [61].

Although ramified microglial cells were previously considered to be in a resting state, a more accurate classification would categorize such a state as one of active surveillance [58,59]. Given that ramified microglial cells are capable of degrading ECM components [62], the increase in the number of such cells in AFL-treated retinas at P17 that we report in this study might represent a crucial process supporting the ordered revascularization of the OIR retina. While P17 is commonly assessed in studies on the OIR model, as it is considered the time-point at which neovascularization reaches its peak [63], we decided to additionally examine the presence of Iba1^+^ cells in OIR retinas at P19 as an early time-point at which the endogenous capacity of the mouse retina to repair its vasculature after the OIR protocol is already at work; full neovascular regression without any therapeutic intervention is routinely observed by P25–P28 [63].

**Figure 4:**
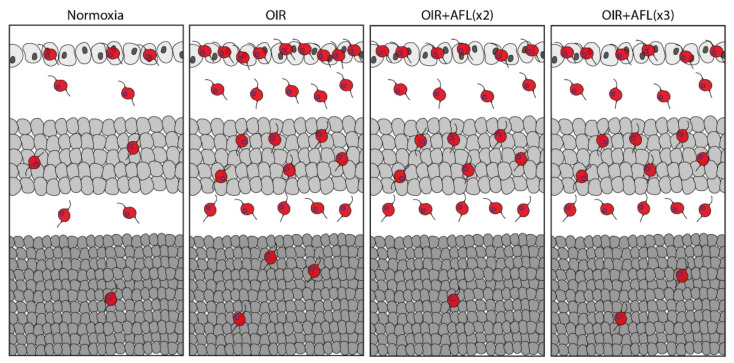
Schematic representation of Iba1+ cells in P19 cross-sections of normoxic, OIR, OIR+AFLx2, and OIR+AFLx3 retinas. Retinal tissue layers are shown in the following order: ganglion cell layer (GCL), top; inner nuclear layer (INL), middle; outer nuclear layer (ONL), bottom.

Previous studies suggest that PlGF signaling via VEGFR-1 is involved in regulating the pathological infiltration and activation of inflammatory cells [64,65]. Indeed, substances targeting only VEGF-A exert no effect on inflammatory activation of microglial/macrophage cells, potentially due to the lack of VEGFR-2 on such cells and to the compensatory upregulation of PlGF upon VEGF inhibition [66,67,68,69,70]. Meanwhile, although PlGF neutralization has been reported to exhibit only mild anti-angiogenic effects in the presence of high VEGF-A levels [71], PlGF blockade might also increase the amount of soluble VEGFR-1 available to trap VEGF-A and thereby indirectly restrict angiogenesis [65,72]. In this sense, AFL might effectively modulate the numbers and activation status of inflammatory cells by simultaneously neutralizing both PlGF and VEGF-A. As AFL has been recently reported to be cleared from the rat’s retina after 48 h [73], we considered that this time period represented a suitable interval between injections. Thus, all mice sacrificed on P17 were injected on P13 and P15. However, mice whose tissues were to be examined on P19 were injected either on P13 and P15 (OIR+AFLx2) or on P13, P15, and P17 (OIR+AFLx3) so as to determine whether animals that had not been treated with AFL for 96 h exhibited any particular changes in the retinal tissue as a consequence of the therapeutic agent being cleared from the system. Unfortunately, our data did not support this hypothesis. Instead, here we report that either two or three doses of AFL lead to a significant reduction in the density of Iba1^+^ cells in the entire OIR retina at P19, as well as to a reduction in the number and density of these cells in the ganglion cell layer also at P19 (Figure 4). While these values remain significantly higher than those found in retinas of animals maintained in normoxic conditions, they provide further evidence for the capacity of AFL to modulate inflammation and, in particular, the behavior of Iba1^+^ microglial cells. As microglia depletion has been shown to impact vascular development in the mouse retina and to reduce neovascularization in CNV and OIR models [19,25,36,74,75], our observations at P19 support the possibility of AFL promoting the clearance of neovascular tufts by modulating microglial cell activity. Thus, regulating the reactivity of microglial cells might be a suitable strategy for the treatment of ischemic retinopathies. In this sense, our results suggest that AFL effectively modulates inflammatory responses in the retina that could be exploited for enhancing its therapeutic efficacy.

## Data Availability

The data presented in this study are available on request from the corresponding authors.
